# Socioeconomic inequalities in disability among older adults in urban and rural Indonesia: a population-based study

**DOI:** 10.1186/s12889-026-27421-1

**Published:** 2026-05-11

**Authors:** Witriani Witriani, Mardiana Dwi Puspitasari, Mugia Bayu Rahardja

**Affiliations:** 1 Center for Psychological Innovation and Research, Faculty of Psychology, Universitas Padjadjaran, Bandung, Indonesia; 2 Department of General, Developmental, and Educational Psychology, Faculty of Psychology, Universitas Padjadjaran, Bandung, Indonesia; 3 Directorate for Human Development, Demography, and Culture Policy, National Research and Innovation Agency (BRIN), Jakarta, Indonesia; 4 Research Center for Population, National Research and Innovation Agency (BRIN), Jakarta, Indonesia

**Keywords:** Household expenditure, Education, Relative index of inequality, Health inequity, Disability, Urban, Rural, Indonesia, Older adults

## Abstract

**Background:**

Disability may restrict older adults’ ability to exercise their rights to health and well-being. The living environment has a significant impact on reducing the risk of disability. Unfortunately, financial insecurity may limit Indonesian older adults’ access to age-friendly services in their environment. This study aims to monitor socioeconomic inequalities in disability, considering both urban and rural contexts.

**Methods:**

The unit of analysis included 129,234 older adults aged 60 and over residing in 514 districts. Data were drawn from the combined datasets of the 2023 National Socioeconomic Survey (SUSENAS) and the 2021 Village Data Census (PODES). Disability status, measured using the Washington Group Six Short questions (WG-SS), was the outcome variable. Socioeconomic Status (SES) was assessed by household expenditure and educational attainment. The Intraclass Correlation Coefficient (ICC) was used to analyze the association between districts and socioeconomic inequalities in disability status. The Relative Index of Inequality (RII) was estimated using log-binomial regression models, adjusting for age, sex, marital status, Self-Rated Health Status (SRHS), and access to primary care facilities and open public space within the district.

**Results:**

Indonesia’s urban and rural areas had ICC values of 22.8%, 20.3%, and 18.9%, respectively. The RII revealed statistically significant differences in disability status. The disparity between the lowest and highest education levels was greater in urban areas (RII = 1.311) than in rural areas (RII = 1.154). Similarly, for household expenditure, the disparity was greater in urban (RII = 1.186) than rural areas (RII = 1.150). As disability is an adverse health outcome, an RII value of more than 1 implies a higher risk of disability among lower SES groups; thus, the risk of disability increases with decreasing SES.

**Conclusions:**

While area development is necessary to minimize the risk of disability, disparities in disability status among older adults with the highest and lowest SES are more pronounced in urban than in rural areas. Addressing issues of person-environment fit is essential to eliminate socioeconomic inequalities in disability status.

## Introduction

Disability can hinder older adults’ ability to fully engage in society and exercise their rights to health and well-being. Globally, the United Nations (UN) estimates that nearly half (over 46%) of older adults aged 60 and above live with a disability, with over 250 million of these individuals experiencing moderate or severe forms [1].

Following the social determinants of health, a cross-country examination has found a positive association between disability and socioeconomic status (SES) among older adults, with disability risk increasing as SES declined [2, 3]. Furthermore, research has indicated that place of residence plays an important role in lowering the risk of disability among older adults [2, 4], as evidenced by the ICF framework, which emphasizes the importance of environment in improving a person’s ability to function, perform, and reduce age-related disability. However, issues of “person-environment fit” should be considered when assessing the age-friendly environments [5, 6].

Research has shown that the features of built environment can make a city to be a suitable place to live and grow old [7]. However, socioeconomically deprived older adults may find it challenging to live in areas where the cost of living is rapidly rising [5]. Instead of providing a convenient means to age in place, urban development may introduce new challenges – such as increased expense and complexity – for socioeconomically deprived older adults. Therefore, this study proposes a refined model of “person-environment fit” specific to the Indonesian context. The analysis will frame the relative index of inequality (RII) generated from the results of multilevel model as evidence of socioeconomic inequalities in disability status. This suggests that development is inadvertently associated with a wider disparity in disability status.

### Indonesia Background

Indonesia’s population is rapidly ageing, with the proportion of older adults aged 60 and over estimated to rise from 9.90% in 2020 to 21.90% in 2050 [8]. Indonesia, much like most Asia-Pacific countries, is witnessing an increase in noncommunicable diseases and age-related disability as its older population continues to grow [9]. Furthermore, many Indonesian older adults live in poverty, with approximately 41.87% of older Indonesians living at the bottom 40% of household purchasing power [10]. Only 20.45% of Indonesian older adults reside at the top 20% of household purchasing power [10].

Prior studies have found a positive association between SES and disability among Indonesian older adults, with the risk of disability increasing as SES declines [11–14]. Furthermore, the living environment plays a significant role in disability status. Living in regions with greatly improved economic and infrastructure development, such as the islands of Java and Bali, as well as urban areas, is associated with a lower risk of disability than living in other regions [11, 12, 15].

However, prior research using a pooled national sample without separately analyzing urban and rural areas may have masked crucial urban-rural differences, potentially concealing a “structural inequality trap” in which development unwittingly widens the socioeconomic inequalities in disability status. This study aims to monitor disparities in disability status based on SES in urban and rural areas, separately, by employing the combined dataset from the 2023 National Socioeconomic Survey (SUSENAS) and the 2021 Village Data Census (PODES) to provide a more nuanced examination of this phenomenon.

### Conceptual review

The World Health Organization (WHO) defines healthy aging as “*the process of developing and maintaining the functional ability that enables well-being in older age*” [16]. Studies have found that poor functional ability poses a risk to well-being among older adults [17, 18]. The 2019 Global Health Estimates reported that mental disorders account for 10.6% of the total disability adjusted life years among older adults aged 60 and older [19].

Healthy aging involves enhancing functional abilities and enabling older adults to engage in meaningful activities, without necessarily being free of illness, injury, or disease [16]. The International Classification of Functioning, Disability, and Health (ICF) defines functional ability as the outcome of the interplay between an individual’s intrinsic capacity and their living environment [20]. The ICF suggests that environments shape what older adults with a certain amount of inherent capacity can be and do [20]. Previous research has shown that services, systems, and policies in the living environment have helped to reduce the risk of disability among Indonesian older adults [11].

The Commission on Social Determinants of Health (CSDH) of the World Health Organization (WHO) believes that health inequities are caused by macrosocial factors operating at the global, national, and local levels—such as governance, macroeconomic policies, social policies, public policies, and culture and societal values—that result in an unfair distribution of health risks and resources to population groups [21, 22]. Under the CSDH conceptual framework, the macro-level social context serves as the fundamental structure of social hierarchy and the socially determined conditions in which people are born, grow, live, work, and age, which results in individuals’ SES [22]. These SES, in turn, shape specific determinants of health as a reflection of people’s social hierarchies, leading to individual differences in exposure and vulnerability to health-threatening situations. Therefore, addressing social determinants of health inequities requires structural adjustments at the macro-level that benefit disadvantaged groups [22, 23].

## Methods

### Data and participants

This cross-sectional study uses a combination of individual- and district-level data. The individual datasets used in the analysis come from the 2023 SUSENAS, notably the March round. The survey employs a two-stage stratified sampling design with a total sample size of 345,000 households (excluding institutional households) spread across 514 districts, using the 2020 Population Census as its master sampling frame. The eligible unit of analysis for this study includes 129,234 household members aged 60 and over, with 53,945 urban and 75,289 rural older adults (unweighted), derived from individual-level data of the 2023 SUSENAS.

The district-level data are from the 2021 PODES. PODES describes infrastructure, social, and economic development at the village level and is conducted three times every ten years. We use the 2021 PODES because it was completed within a close time interval to the 2023 SUSENAS. It includes 84,096 urban and rural villages organized into 514 districts.

### Measures

#### Outcome variable

The outcome variable of this study is disability. Disability is assessed employing the Washington Group Six Short questions (WG-SS), a tool developed by the UN Washington Group on Disability Statistics. These questions align with those in the 2023 SUSENAS. The WG-SS covers six domains of activity: seeing, hearing, walking or climbing steps, remembering or concentrating, self-care, and communicating. Each domain is rated on an ascending difficulty scale: “no difficulty,” “some difficulty,” “a lot of difficulty,” and “cannot do at all.” Following the cut-off point established by the United Nations International Seminar on the Measurement of Disability, we define disability as having “some difficulty” in at least two of the six domains, or “a lot of difficulty” or “cannot do at all” in at least one domain [11, 12, 24, 25]. This threshold has been documented in several studies conducted in other countries, including India, Malaysia, Palestine, Peru, and South Africa [26–30]. We selected this cut-off point to increase sensitivity [31, 32]. On the other hand, only 9.36% of older adults with disability follow the more conservative WG recommendation, indicating “a lot of difficulty” or “cannot do at all” in at least one domain.

#### Variables of interest

##### Household expenditure

Household consumption is often the core concept in efforts to quantify living standards, inequality, and poverty in developing countries [33]. Statistics Indonesia (*Badan Pusat Statistik* [BPS]) calculates household expenditure using the basic need approach, which includes total expenditure on food and non-food items. Households are then divided into three groups based on their expenditure, following World Bank criteria: the bottom 40%, the middle 40%, and the top 20% [34]. The household expenditure is calculated annually using the full sample of SUSENAS households, with updated household listings made prior to the survey.

##### The highest level of education

Educational attainment is categorized into three levels: low (primary school or lower), medium (middle and high school), and high (university education). Since 2015, all Indonesian citizens have been required to complete twelve years of compulsory education, which includes six years of primary school, three years of middle school, and three years of high school. Thus, in our analysis, the medium level of educational accomplishment is set at the 2023 average of 8.77 years of schooling, which corresponds to middle and high school levels [35].

#### Control variables

We included control variables at both individual and environmental levels in our analysis. Due to data limitations and the cross-sectional survey design, we are unable to identify several possible confounders, including life course trajectory and household composition [36–38]. Otherwise, we include marital status, which might be regarded part of the household composition. We also identified urban and rural areas as components of geographic location that may improve functional health. According to BPS regulation No. 120/2020 on Urban- and Rural-Villages Classification in Indonesia, infrastructure developments determine the official typology of urban and rural areas.

1. Individual-level Controls
  2. Age: Categorized into five groups: 60-64, 65-69, 70-74, 75-79, and 80+ years.
  3. Sex: Classified as male or female.
  4. Marital status: Grouped as currently married, divorced, or widowed.
  5. Self-Rated Health Status (SRHS): This variable was coded as yes/no. SRHS is a simple, self-reported measure of perceived health, widely used in health and social research, and acknowledged by the WHO [39]. Typically, it allows individuals to rate their current health status on a 5-point Likert scale (ranging from very good to very bad) [39]. As the 2023 SUSENAS lacked a five-point Likert scale for self-perceived health, we operationalized Self-Rated Health Status (SRHS) as a dichotomous variable (yes/no). This was based on the question about self-perceived poor health within one month, such as experiencing flu, fever, diarrhea, chronic disease and so on, within one month of the survey.
2. Environmental-level Controls
  7. Percentages of urban and rural villages in the district with public primary care facilities: This includes primary health centers, clinics, and hospitals. Primary care is highly important in Indonesia, as it provides comprehensive services ranging from health promotion to palliative care, manages chronic illnesses, and offers outpatient treatment, all of which can be covered by government-funded health insurance. Receiving outpatient treatment at primary care facilities is particularly crucial, as these serve as the first point of referral for further government-funded healthcare services such as specialist consultations and inpatient care [40, 41].
  8. Percentages of urban and rural villages in the district with open public spaces: For the purpose of data collection, BPS defines open public space as public land that is primarily intended for leisure activities, such as playing and community gathering, with free admission. Open public space includes fields, squares, parks, playgrounds, etc.

It is important to note that both public primary care facilities and open public spaces are designed to assist all socioeconomic groups, including those in a higher SES. However, not every village has public primary care services or open public spaces.

### Statistical analysis

First, we always include the survey design and sampling weight of the 2023 SUSENAS in our analysis. Sampling weights from SUSENAS 2023 were applied at the individual level in all analyses to account for the complex survey design. In the multilevel models, individual-level weights were incorporated to obtain population-representative estimates. Given the absence of explicit district-level sampling weights, district-level variables were treated as contextual covariates without additional weighting. All analyses were conducted using SPSS Complex Samples procedures, which supports the integration of survey weights in regression modelling. We first collapsed the 2021 PODES village-level data into district-level aggregates, calculating the percentage of villages equipped with healthcare facilities and open public spaces within each district that were classified as urban and rural areas based on the official BPS typology provided in the dataset. These district-level aggregates were then integrated with individual-level 2023 SUSENAS data, using district identifiers as the primary linkage key. All individual-level SUSENAS respondents were assigned the district-level characteristics. This approach assumes that district-level infrastructure characteristics (healthcare facilities and open public spaces) from 2021 remained relatively stable through 2023, which is reasonable given that large-scale infrastructure development typically occurs over longer time horizons. The numerical descriptions of categorical variables are expressed as percentages (n=%).

Second, the intraclass correlation coefficient (ICC) was calculated from the null model to quantify the proportion of variance attributable to between-district differences. Multilevel logistic regression models were used to estimate adjusted odds ratios (ORs) of disability, examining the association between SES and disability status while controlling for individual and district-level factors. We also address geographical differences by analyzing separate models for urban and rural areas. Urban–rural stratified analyses were conducted to explore differences in socioeconomic gradients of disability across contextual settings.

Third, the Relative Index of Inequality (RII) is computed to further estimate the relative disparities in disability status between older adults across the socioeconomic spectrum. The RII was estimated separately for educational attainment and household expenditure using log-binomial regression models with SES ridit rank as a continuous predictor. Ridit scores were constructed based on the weighted cumulative distribution of SES categories, following standard methods for health inequality measurement [42, 43]. The RII models were adjusted for individual-level covariates and district-level characteristics and were estimated separately for urban and rural populations. These models were estimated separately from the multilevel logistic regression analyses, which were used to assess contextual variation in disability.

The RII was chosen as a robust, regression-based measure to quantify socioeconomic disparities, as it accounts for the population’s distribution across socioeconomic status (SES) categories and allows for comparisons between different SES indicators. As disability is an adverse health outcome, an RII value less than 1 would imply a lower risk of disability among lower socioeconomic status (SES) groups, indicating the outcome is concentrated among the most disadvantaged [44]. These indices account for the proportion of people in the different SES categories and show the relative risk of disability when comparing the lowest to the highest categories, regardless of the number of categories or their distribution across the sample. To ensure the robustness of our findings in a large sample, we adjusted for a standardizing covariate in our multilevel models to account for the effects of other variables on the RII. We also constructed the RII’s confidence intervals using regression-based standard errors to mitigate bias and improve the precision of our estimates [45].

### Data availability statement

The dataset for this study is accessible through a government-to-government agreement between BPS and BRIN. The agreement imposes legal restrictions that prevent the data from being shared publicly. We obtained permission to use the dataset from BRIN’s Center for Data and Information via official letters number B-2446/III.7.3/DI.00/9/2023 and B-7055/III.9.3/TK.02.02/5/2024. BPS provides anonymized datasets to BRIN. Therefore, the data obtained is in compliance with data protection regulations.

## Results

The percentage of total participants with disability is approximately 21.86% (Table 1). Rural areas have a larger percentage of participants with disability (23.48%) than urban areas (20.56%). In addition, the percentage of villages in the district with public primary care facilities is greater in urban (66.75%) than rural areas (48.67%). Similarly, the percentage of villages in the district with open public space is greater in urban (46.99%) than rural areas (32.28%).

**Table 1:** Basic characteristics of study population

Characteristics	Urban		Rural		Total	
	*n*	% weighted (95% CI)	*n*	% weighted (95% CI)	*n*	% weighted (95% CI)
Individual-level						
Disability status						
No	43,383	79.44 (79.42–79.46)	56,793	76.52 (76.49–76.54)	100,176	78.14 (78.12–78.15)
Yes	10,562	20.56 (20.54–20.58)	18,496	23.48 (23.46–23.51)	29,058	21.86 (21.85–21.88)
Age group						
60–64	22,254	37.36 (37.34–37.38)	29,882	35.65 (35.63–35.67)	52,136	36.60 (36.58–36.61)
65–69	14,769	27.19 (27.17–27.21)	19,479	26.74 (26.72–26.76)	34,248	26.99 (26.98–27.01)
70–74	8,817	17.82 (17.81–17.84)	12,877	18.35 (18.33–18.37)	21,694	18.06 (18.05–18.07)
75–79	4,398	9.61 (9.60–9.62)	6,806	9.81 (9.80–9.83)	11,204	9.70 (9.69–9.71)
80+	3,707	8.01 (8.00-8.03)	6,245	9.45 (9.43–9.46)	9,952	8.65 (8.64–8.66)
Sex						
Male	26,051	47.65 (47.63–47.67)	36,959	47.80 (47.77–47.82)	63,010	47.72 (47.70-47.73)
Female	27,894	52.35 (52.33–52.37)	38,330	52.20 (52.18–52.23)	66,224	52.28 (52.27–52.30)
Marital status						
Currently married	33,558	64.09 (64.07–64.12)	48,562	66.68 (66.66–66.71)	82,120	65.25 (65.23–65.27)
Single	935	1.09 (1.08–1.09)	990	0.94 (0.93–0.94)	1,925	1.02 (1.01–1.03)
Divorced	1,393	2.05 (2.04–2.05)	1,621	1.80 (1.80–1.81)	3,014	1.94 (1.93–1.94)
Widowed	18,059	32.77 (32.75–32.79)	24,116	30.58 (30.56–30.60)	42,175	31.79 (31.78–31.81)
Self-rated health status (SRHS)						
Yes	22,027	41.24 (41.22–41.26)	32,136	41.81 (41.78–41.83)	54,163	41.49 (41.48–41.51)
No	31,918	58.76 (58.74–58.78)	43,153	58.19 (58.17–58.22)	75,071	58.51 (58.49–58.52)
Education						
Low	31,693	63.55 (63.52–63.57)	62,127	84.95 (84.93–94.97)	93,820	73.10 (73.09–73.12)
Medium	16,296	26.59 (26.57–26.61)	10,662	12.12 (12.10-12.13)	26,958	20.13 (20.12–20.14)
High	5,956	9.86 (9.85–9.87)	2,500	2.93 (2.93–2.94)	8,456	6.77 (6.76–6.78)
Household expenditure						
Bottom 40%	22,571	48.53 (48.51–48.55)	33,040	50.63 (50.61–50.66)	55,611	49.47 (49.45–49.49)
Mid 40%	20,380	34.20 (34.18–34.22)	29,313	35.87 (35.84–35.89)	49,693	18.56 (18.54–34.96)
Top 20%	10,994	17.27 (17.25–17.28)	12,936	13.50 (13.48–13.52)	23,930	15.59 (15.57–15.60)
District-level						
Percentage of villages with primary care services; mean (SD)	66.75 (23.07)		48.67 (18.94)		56.22 (23.00)	
Percentage of villages with open public space; mean (SD)	46.99 (23.23)		32.28 (21.32)		38.42 (23.15)	

Table 2 shows that the random intercept variance in total, urban, and rural are significant at α = 0.05, suggesting significant variance among districts in the model. Furthermore, the ICC values for total, urban, and rural areas are all greater than the 0.05 (or 5%) criteria, implying that differences in characteristics among districts can explain disparities in disability status. These two statistical analyses confirm that district characteristics influence disability status in participants. Specifically, variations in district-level characteristics account for approximately 22.8%, 20.3%, and 18.9% of the disparities in disability status in Indonesia (total), urban, and rural areas, respectively.

**Table 2:** Multilevel logistic regression: Odds ratio of disability status

Characteristics	Urban				Rural				Total		
	OR	*p*-value*.	95% CI for OR	OR		*p*-value *.	95% CI for OR	OR		*p*-value *.	95% CI for OR
Individual-level											
Age group											
60–64	1.00			1.00				1.00			
65–69	1.68	0.00	1.58–1.78	1.54		0.00	1.47–1.62	1.59		0.00	1.53–1.65
70–74	2.77	0.00	2.59–2.96	2.64		0.00	2.51–2.78	2.69		0.00	2.59–2.81
75–79	4.92	0.00	4.55–5.32	4.26		0.00	4.01–4.52	4.51		0.00	4.30–4.74
80+	9.45	0.00	8.71–10.26	7.14		0.00	6.70–7.61	7.97		0.00	7.58–8.38
Sex											
Male	1.00			1.00				1.00			
Female	1.18	0.00	1.12–1.24	1.20		0.00	1.15–1.25	1.19		0.00	1.15–1.23
Marital status											
Currently married	1.00			1.00				1.00			
Single	1.54	0.00	1.30–1.82	1.96		0.00	1.77–2.11	1.85		0.00	1.67–2.08
Divorced	1.25	0.04	1.00–1.55	1.37		0.00	1.13–1.63	1.30		0.00	1.13–1.50
Widowed	1.35	0.02	1.14–1.60	1.46		0.00	1.23–1.68	1.41		0.00	1.21–1.65
Self-rated health status (SRHS)											
Yes	1.00			1.00				1.00			
No	0.49	0.00	0.46–0.51	0.50		0.00	0.48–0.52	0.49		0.00	0.48–0.51
Education											
Low	1.00			1.00				1.00			
Medium	0.82	0.00	0.78–0.87	0.92		0.01	0.87–0.97	0.84		0.00	0.81–0.87
High	0.70	0.00	0.64–0.76	0.80		0.00	0.72–0.90	0.71		0.00	0.67–0.76
Household expenditure											
Bottom 40%	1.00			1.00				1.00			
Mid 40%	0.81	0.00	0.75–0.86	0.90		0.00	0.84–0.94	0.88		0.00	0.83–0.90
Top 20%	0.76	0.00	0.71–0.83	0.78		0.00	0.75–0.84	0.72		0.00	0.69–0.75
District-level											
Percentage of villages with primary care services	1.00	0.68	0.99–1.01	0.98		0.01	0.98–0.99	0.98		0.01	0.98–0.99
Percentage of villages with open public spaces	0.98	0.00	0.97–0.99	0.95		0.00	0.94–0.94	0.94		0.00	0.93–0.95
Constant	0.31	0.00		0.54		0.00		0.46		0.00	
Random intercept variance ** (${{\upsigma}}_{\mathrm{u}}^{2}$)	0.836			0.769				0.975			
ICC**	0.203			0.189				0.228			

^*^all the explanatory variables are statistically significant at α = 0.05

^**^random intercept variance and ICC are developed without allowing for explanatory variables to be inserted into the model (the null model)

After controlling for age, sex, marital status, SRHS, and the percentages of urban and rural villages in the district with primary care facilities and open public space (Table 3), the RII indicates significant findings. Specifically, the relative disparities in disability status between the lowest and highest education levels is greater in urban areas (RII = 1.311) than in rural areas (RII = 1.154). This consistently shows a higher risk of disability among individuals with lower education in both settings (RII > 1). Furthermore, the RII also reveals that the relative disparity in disability status between the lowest and highest household expenditure groups is greater in urban areas (RII = 1.186) compared to rural areas (RII = 1.150). Here too, a higher risk of disability is observed among those with lower household expenditure (RII > 1).

**Table 3:** Relative inequalities in disability status by urban, rural, and total

	Education			Household expenditure		
Relative inequalities	RII	95%CI	*p*-value	RII	95%CI	*p*-value
Urban	1.311	(1.216; 1.421)	0.000	1.186	(1.118; 1.255)	0.000
Rural	1.154	(1.069; 1.248)	0.000	1.150	(1.107; 1.193)	0.000
Total	1.274	(1.210; 1.350)	0.000	1.149	(1.113; 1.183)	0.000

^*^all the explanatory variables are statistically significant at α = 0.05

## Discussion

Our study finds significant disparities in disability status among Indonesian older adults based on SES, with the risk of disability increasing as socioeconomic disadvantage rises. Furthermore, disparities in disability status between the highest and lowest of the household expenditure and education distributions persist.

The descriptive analysis shows that 73.1% of older adults had only primary education or less, with only 6.77% having a university education. Many studies have highlighted the critical role of early life experiences in predicting various health outcomes in old age by providing access to a variety of material and non-material resources, such as income, employment, safe neighborhoods, and healthy lifestyles. Education improves one’s knowledge, skills, health literacy, and a wide range of other capabilities, all of which influence health status outcomes later in life [46–50].

Striking urban-rural differences exist, with more urban older adults having higher education and fewer urban older adults having lower education than those in rural areas (see Table 1). Low-SES older persons in rural areas experience a ‘double disadvantage’ in that they not only have fewer years of schooling but also live in locations with less infrastructure to compensate for lower health literacy and fewer economic possibilities throughout their lives.

We find larger education inequalities for disability status among older adults in urban (RII = 1.311) than in rural (RII = 1.154) areas. The larger educational inequality in urban areas compared to rural areas may indicate greater educational heterogeneity in urban areas, allowing for more evident divergence of health outcomes across the education spectrum. In rural areas, where the population is more educationally homogenous (84.95% with low education), the reduced variability may attenuate observable inequalities. Although our study found that older adults with lower education were more likely to have a disability than those with higher education, macro-level interventions must benefit all older adults due to their risk of cognitive decline as they age. Regardless of their early-life education level, older adults are at risk of low health literacy, which is associated with increasing age and cognitive deterioration [47, 51].

The use of multilevel analysis yielded precise estimates of the fixed effects of place of residence and individual factors on disability status, the variability in disability status across districts, and the extent to which individual factors could explain observed geographical inequalities in disability status. As evidenced by the ICC values, place of residence plays an important role in disability status among older adults. Thus, we argue that development may unwittingly contribute to the socioeconomic inequalities in disability status.

In addition, we performed multilevel analyses separately for urban and rural areas. We find that ICC values in urban areas are slightly higher than in rural areas, implying that urban development has a greater impact on disability status among urban older adults than rural development has on disability status among rural older adults. Supply-side factors in the place of living, such as primary care facilities and open public spaces, have a slightly greater impact on reducing the risk of disability status in urban villages than in rural villages.

Healthcare services and facilities in Indonesia remain relatively unequally distributed across urban and rural areas. Recent studies found that rural primary health centers often suffer from critical shortages of healthcare workforces [52, 53], healthcare service interventions [54], and essential medicines [55]. Furthermore, poorer rural infrastructures may provide insurmountable challenges for older adults without adequate supports.

On the other hand, this study finds socioeconomic inequalities in disability status are greater in urban than rural areas. While urban development, including the availability of primary care services and open public spaces, offers more opportunities to lower the risk of disability, it is also associated with a widening of socioeconomic disparities in disability status, creating a “structural inequality trap”, when compared to rural areas. Even when primary care services and open public spaces in urban areas are developed with the intention of benefiting all older adults, low-SES older adults may accidentally be excluded from these services, making low-SES urban older adults more susceptible to disability. Unfortunately, our study cannot assess the qualitative aspects of primary care services and open public spaces, such as quality, accessibility, and affordability, which would allow us to conduct a more in-depth examination into the exclusion of low-SES older adults from these services and spaces.

Along with the implementation of Universal Health Coverage (UHC) in 2014, a recent study found that public primary care services had reached all districts in Indonesia; however there were only a few referral hospitals with complete facilities available [56]. Public primary care services were mainly used by the socioeconomically disadvantaged population and those living in underdeveloped areas [57, 58]. Underdeveloped areas frequently lack referral hospitals with complete facilities [59]. With limited healthcare facilities options in underdeveloped areas, older adults of all SES may use public primary care services, lowering the socioeconomic inequality in disability status. On the other hand, as a village’s economic development stage advances, high-SES persons are less likely to use public primary care services and prefer to go directly to private healthcare providers [58]. Private healthcare providers typically have more comprehensive facilities and require more out-of-pocket expenses. The findings may explain why socioeconomic inequalities in disability status are lower in rural than urban areas.

Open public space is primarily intended for all socioeconomic groups to engage in physical and psychosocial activities. Aside from physical infrastructure, open public space serves as social infrastructure, promoting an inclusive environment and social connections. Prior research in rural villages found that ‘bale desa’ (a public open space) served as a central location for rural communities to gather, coordinate, and show unity [46, 47]. In contrast, other studies have revealed certain accessibility challenges for open public spaces, particularly in urban settings.

Kurniawan HB and Raychansyah MS, for example, discovered that elite private neighborhoods were closer to open green public spaces than undeserved neighborhoods [60]. Other studies have revealed that the development of social facilities in metropolitan areas is limited in wealthier areas [61, 62]; thus, low-SES individuals may be more vulnerable in terms of accessibility and affordability. Findings from both rural and urban research on access to open public space may help explain the mechanism of disparity in urban areas, thereby contributing to a better understanding of the ‘structural inequality trap’.

### Limitations

This study uses a nationally representative household survey, the 2023 SUSENAS, which includes both health outcomes (disability status) and data characterizing specific dimensions of inequity, such as wealth, education, region, and sex. This makes it a relevant data source for monitoring health inequity, as recommended by WHO [43, 63]. WHO recommends monitoring health inequity, particularly in LMICs [43]. Monitoring health inequity only reflects how people live their lives today and cannot directly answer whether a policy had an impact, but it can assist policymakers in determining whether a change is necessary [43, 63].

However, some limitations remain. First, the 2023 SUSENAS data only includes non-institutionalized older adults, our findings may not be generalizable to the institutionalized population, which is expected to have a higher prevalence of disability. Second, disability status is self-reported and may be subject to social desirability or literacy bias, which could be particularly prominent among low-SES older adults. Third, data on household expenditure may be susceptible to recall bias. This is because the respondents are asked to recall food expenditures over the last week and non-food expenditures over the past year. Fourth, the 2021 PODES data only reports on the presence of facilities, such asprimary care services and open public spaces in villages, thereby weakening the link between the conceptual framework of ‘person-environment fit’ and the empirical model. This does not fully capture the qualitative aspects of these facilities, such as quality, accessibility, and affordability, which are essential for a comprehensive assessment of ‘person-environment fit’. Finally, while we anticipate that large-scale infrastructure development normally takes place over longer time periods, we acknowledge that a temporal lag between the 2023 individual-level and 2021 district-level covariates may weaken or distort associations.

## Conclusion

This study finds that significant disparities in disability status among Indonesian older adults in the highest and lowest SES persist, with disability status increasing as socioeconomic disadvantage increased. Because place of residence plays an important role in disability status among older adults, development may unwittingly contribute to the socioeconomic inequalities in disability status.

In addition, urban development has a greater influence on lowering the risk of disability among urban older adults than rural development does on rural older adults. However, socioeconomic inequalities in disability status are greater in urban than rural areas. While urban development, including the availability of primary care services and open public spaces, provides more opportunities to reduce the risk of disability, it is also associated with a widening of socioeconomic inequalities in disability status, creating a “structural inequality trap”.

Our study calls for macro-level interventions, including urban planning, fiscal policy, and a stronger social protection system. Urban development must create an inclusive environment by making public spaces more accessible and affordable and enhancing the quality of public primary care. We also propose targeted fiscal policies: subsidies for older adults to access age-friendly urban services and subsidies for public primary care facilities across Indonesia to provide comprehensive preventive and rehabilitative care. Ultimately, we advocate for strengthening the social protection system, as the majority of older adults live in poverty and face a high risk of poor health and disability.

Given the limitations of our study, we recommend directions for future investigations. We did not examine causality in our study because it is designed to monitor health inequity by measuring socioeconomic inequalities in disability status. Therefore, future research can use mixed-methods studies that combine quantitative data and qualitative insights to establish causality and investigate the ‘why’ of observed inequality. Future study can also look at time trends to see if current inequities have improved or deteriorated, which can assist policymakers decide whether a change is necessary. Policy-oriented research is strongly recommended to provide greater benefit since it assesses the effectiveness of specific actions or policies in improving person-environment fit.

## Data Availability

The dataset for this study is accessible through a government-to-government agreement between BPS and BRIN. The agreement imposes legal restrictions that prevent the data from being shared publicly. We obtained permission to use the dataset from BRIN’s Center for Data and Information via official letters number B-2446/III.7.3/DI.00/9/2023 and B-7055/III.9.3/TK.02.02/5/2024. BPS provides anonymized datasets to BRIN. Therefore, the data obtained is in compliance with data protection regulations.

## Abbreviations

BPS: Statistics Indonesia (Badan Pusat Statistik)
CSDH: Commission on Social Determinants of Health
ICC: Intraclass Correlation Coefficient
LMICs: Low- and Middle-Income Countries
PODES: Village Data Census
RII: Relative Index of Inequality
SES: Socioeconomic Status
SRHS: Self-Rated Health Status
SUSENAS: National Socioeconomic Survey
WG-SS: Washington Group Six Short questions
